# Supplementary material for: Development of a computer-aided design software for the quantitative evaluation of aesthetic damage
Source: PLoS One. 2019 Dec 18;14(12):e0226322. doi: 10.1371/journal.pone.0226322 (PMC6919621; doi:10.1371/journal.pone.0226322)
Supplement: S1 Code site — 1. (ZIP) [file pone.0226322.s001.zip › [EESC_jr][Projeto]Relatorio_07_21/vendors/select2/docs/_includes/options/dropdown/selections.html]

## Can I change how selecting results works?

### Can I select the highlighted result when the dropdown is closed?

{% highlight js linenos %}
$('select').select2({
selectOnClose: true
});
{% endhighlight %}
{% include options/not-written.html %}

### Can I prevent the dropdown from closing when a result is selected?

{% highlight js linenos %}
$('select').select2({
closeOnSelect: false
});
{% endhighlight %}
{% include options/not-written.html %}
